# Revisiting the association between human leukocyte antigen and end-stage renal disease

**DOI:** 10.1371/journal.pone.0238878

**Published:** 2020-09-11

**Authors:** Naila Noureen, Farhad Ali Shah, Jan Lisec, Hina Usman, Mohammad Khalid, Rimsha Munir, Nousheen Zaidi

**Affiliations:** 1 Cancer Biology Lab, MMG, University of the Punjab, Lahore, Pakistan; 2 Cancer Research Centre (CRC), University of the Punjab, Lahore, Pakistan; 3 Bundesanstalt für Materialforschung und -prüfung (BAM), Department of Analytical Chemistry, Berlin, Germany; 4 MMG, The Women University Multan, Multan, Pakistan; 5 Hormone Lab, Lahore, Pakistan; Peking University First Hospital, CHINA

## Abstract

Multiple works have studied possible associations between human leukocyte antigen (HLA) alleles and end stage renal disease (ESRD) showing, however, contradictory and inconsistent results. Here, we revisit the association between ESRD and HLA antigens, comparing HLA polymorphism (at HLA-A, -B, -C, -DRB1, -DQB1 and DQA1 loci) in ESRD patients (n = 497) and controls (n = 672). Our data identified several HLA alleles that displayed a significant positive or negative association with ESRD. We also determined whether heterozygosity or homozygosity of the ESRD-associated HLA alleles at different loci could modify the prevalence of the disease. Few HLA allele combinations displayed significant associations with ESRD, among which A*3_26 combination showed the highest strength of association (OR = 4.488, P≤ 0.05) with ESRD. Interestingly, the age of ESRD onset was not affected by HLA allele combinations at different loci. We also performed an extensive literature analysis to determine whether the association of HLA to ESRD can be similar across different ethnic groups. Our analysis showed that at least certain HLA alleles, HLA-A*11, HLA-DRB1*11, and HLA-DRB1*4, display a significant association with ESRD in different ethnic groups. The findings of our study will help in determining possible protective or susceptible roles of various HLA alleles in ESRD.

## Introduction

A wide array of research works have indicated associations between human leukocyte antigen (HLA) status and various kidney diseases [1]. Few of these disorders are immune-mediated, while, in others, the pathogenesis is unclear or the relevance of HLA is not entirely understood. The end-stage renal disease (ESRD)–last stage of chronic renal failure–has become a global health problem [2] and has been investigated for HLA association [3–5]. In the majority of the previous works, ESRD patients waiting for kidney transplantation were examined. Nonetheless, there are several contradictions in these studies, and no consistent trend on the association between different HLA alleles and ESRD was observed [1]. It has been suggested that a few HLA alleles may affect the severity of kidney disease or risk of progression [1]. For instance, specific HLA alleles could promote a more generalized pro-fibrogenic T cell phenotype that may contribute to disease progression or onset [1].

The presented study aims to revisit the association between HLA-polymorphism and ESRD. Most of the previous works have studied this association in homogeneous ethnic groups, and they have interpreted their data accordingly. For this study, we compare the HLA polymorphism (at HLA-A, -B, -C, -DRB1, -DQB1 and DQA1 loci) in ESRD patients and controls from Punjab–one of the better-developed regions in Pakistan. To the best of our knowledge, none of the previous works have studied the association between HLA alleles and ESRD in the Pakistani population. Also, only a few earlier works have studied HLA allele frequencies in the healthy Pakistani population [6–9] (**S1 Table**). These studies also have limitations with respect to the population size [6, 7] or the limited number of HLA loci studied. Here, we try to overcome these limitations by examining the broader-array of HLA genes in a larger population sample. Previous works have shown that for certain pathological conditions, heterozygosity, or homozygosity of the disease-associated HLA alleles could modify the manifestation of that condition [10]. Hence, here we aimed to determine whether heterozygosity or homozygosity of the ESRD-associated HLA alleles at different loci could modify the prevalence of the disease. Moreover, we also compared our data with previous significant studies to identify any consistent patterns of HLA allele associations with ESRD. The findings of this study will help in determining possible protective or susceptible roles of various HLA alleles in ESRD.

## Materials and methods

### Study-population, ethics and sample collection

For the present study, we examined the ESRD patients (n = 497) that were waitlisted (from 2017–2019) for renal transplantation at various transplant centers across Punjab, Pakistan. In addition, healthy subjects (n = 672) from the same region were also included in this study as a control population. All the study subjects were above the age of 15. We excluded subjects with the following conditions: severe viral/bacterial infection, on anticoagulation therapy, suffering from bleeding disorder (e.g. hemophilia, low platelets, etc.) and aplastic anemia. The study protocol for human subjects was approved by the Research Ethics and Biosafety Committee-MMG, University of the Punjab. Informed consent was obtained from each study-subject before sample collection. To obtain the basic personal information and medical history each participant was interviewed and completed a structured questionnaire. The medical history file of each patient was also thoroughly examined. Intravenous blood was collected from all the subjects according to the guidelines of the National Committee for Clinical Laboratory Standards (document H18-A4) [11] in vials containing EDTA-anticoagulant agent.

### DNA extraction and HLA genotyping

Genomic DNA was extracted from whole blood using DNAZOL BD reagent according to the manufacturer’s instructions (Thermofisher Scientific). DNA concentration and purity were determined by Nanodrop spectrophotometer (ThermoScientific, USA). The DNA samples were stored at −20°C until use. The samples were typed for HLA-A, -B, -DRB1, -DQB1,-DQA1 loci by sequence-specific oligonucleotide PCR (SSO-PCR) using LIFECODES HLA SSO typing kits. For HLA-C typing LIFECODES HLA-C eRES SSO typing kits were used. The product signals were detected by Luminex-200 and XY platform and were analyzed by MatchIT DNA software (Immucor GTI diagnostics inc. USA) according to the instructions mentioned in the software manual.

### Statistical analysis

HLA-A, -B, -C, -DRB1,-DQB1, and DQA1 allele frequencies (AF) were determined for each allele in patients with ESRD and controls using the following formula: AF (%) = (n/2N)×100, where n indicated the sum of a particular allele and N indicated the total number of individuals. The differences in allele percentages between patients with ESRD and controls were analyzed by cross-tabulation using the Fisher test. In the statistical process, P-value was calculated according to the expected value. The strength of disease association to a particular allele was expressed by odds ratio (OR) at 95% confidence intervals (95% CI). Statistical significance was accepted when P < 0.05. Alleles with OR >1.00 were considered to be positively associated with ESRD. Alleles with OR < 1.00 were considered to be negatively associated with ESRD. Analysis of allele combinations was performed using the R software environment 3.4.2 (http://cran.r-project.org/).

## Results

### HLA-A, -B, -C, -DRB1, -DQB1 and DQA1 allele frequencies: ESRD patients vs. controls

The common alleles (percentage frequency >0.1%) identified at HLA-A, -B, -C, -DRB1, -DQB1 and DQA1 loci in ESRD patients or control population are listed in **Table 1**. We compared the percentage frequencies of these alleles between the end-stage renal disease (ESRD) patients and the control group.

**Table 1:** Percentage frequencies for HLA alleles in control and ESRD patients.

Locus	Allele	Controls (% frequency)	ESRD (% frequency)	OR	95% CI (0R)	P- Value
**A**	A*02	15.92	14.49	0.708	0.5548–0.904	* *
**A**	A*11	14.88	13.98	0.930	0.736–1.1749	* *
**A**	A*01	13.76	12.88	0.918	0.7201–1.1694	* *
**A**	A*24	11.46	10.97	0.952	0.7336–1.2347	* *
**A**	A*26	9.97	11.27	1.135	0.8699–1.4812	* *
**A**	A*68	6.40	8.08	1.280	0.9333–1.7564	* *
**A**	A*03	6.18	7.14	1.169	0.8418–1.6224	* *
**A**	A*33	6.47	5.84	0.895	0.6354–1.2614	* *
**A**	A*31	5.28	4.63	0.870	0.5947–1.2727	* *
**A**	A*32	4.24	4.43	1.046	0.6995–1.5635	* *
**A**	A*30	1.71	2.72	1.604	0.9139–2.814	* *
**A**	A*29	1.71	1.71	0.999	0.531–1.8808	* *
**A**	A*23	0.89	0.91	1.014	0.4257–2.4165	* *
**A**	A*74	0.60	0.81	1.355	0.5068–3.6227	* *
**A**	A*66	0.15	0.10	0.676	0.0612–7.4629	* *
**A**	A*36	0.30	0.00			* *
**A**	A*66	0.15	0.10			* *
**A**	A*34	0.00	0.10			* *
**B**	B*08	13.84	14.69	1.072	0.8482–1.3547	* *
**B**	B*40	14.21	11.07	0.751	0.5848–0.9649	**p ≤ 0.05**
**B**	B*35	11.16	11.57	1.041	0.8045–1.348	* *
**B**	B*51	10.49	9.15	0.860	0.6516–1.1346	* *
**B**	B*52	6.85	6.94	1.015	0.7346–1.4029	* *
**B**	B*15	6.32	5.33	0.834	0.586–1.1877	* *
**B**	B*44	4.39	4.93	1.129	0.7661–1.6647	* *
**B**	B*07	4.39	4.53	1.129	0.7661–1.6647	* *
**B**	B*57	4.76	3.92	0.817	0.5437–1.2269	* *
**B**	B*50	3.20	4.93	1.569	1.0328–2.383	**p ≤ 0.05**
**B**	B*13	3.35	3.42	1.022	0.6499–1.6084	* *
**B**	B*58	3.20	2.92	0.210	0.1291–0.3428	* *
**B**	B*37	2.38	3.12	1.320	0.7998–2.178	* *
**B**	B*55	2.16	3.32	1.557	0.939–2.5821	* *
**B**	B*27	2.23	2.72	1.223	0.7223–2.0705	* *
**B**	B*18	1.56	1.41	0.900	0.4554–1.7788	* *
**B**	B*38	1.19	1.21	1.014	0.4777–2.1537	* *
**B**	B*41	0.97	1.21	1.251	0.5684–2.7539	* *
**B**	B*39	0.89	1.11	1.242	0.5458–2.8267	* *
**B**	B*45	0.67	0.91	1.355	0.536–3.4269	* *
**B**	B*56	0.52	0.40	0.772	0.22530–2.6435	* *
**B**	B*53	0.22	0.60	2.715	0.6772–10.8808	* *
**B**	B*49	0.37	0.30	0.811	0.1933–3.4003	* *
**B**	B*48	0.22	0.10	0.450	0.0468–4.3342	* *
**B**	B*14	0.15	0.00			* *
**B**	B*42	0.15	0.00			* *
**B**	B*68	0.00	0.10			* *
**C**	C*07	26.53	30.05	1.135	0.5806–2.2174	* *
**C**	C*12	15.82	13.79	1.924	0.4283–8.6434	* *
**C**	C*06	14.80	13.55	1.382	0.8379–2.2786	* *
**C**	C*15	11.90	11.58	1.049	0.6837–1.6107	* *
**C**	C*04	9.35	9.85	1.151	0.3071–4.3108	** **
**C**	C*03	5.78	7.88	0.894	0.6209–1.2862	* *
**C**	C*16	3.40	3.94	1.176	0.8885–1.556	* *
**C**	C*01	3.91	2.71	0.551	0.2279–1.3312	* *
**C**	C*14	3.06	2.71	0.843	0.5892–1.2071	* *
**C**	C*08	3.06	1.72	0.874	0.4085–1.8714	* *
**C**	C*05	0.85	0.99	0.960	0.6476–1.4222	* *
**C**	C*02	0.51	0.99	0.678	0.3269–1.4073	* *
**C**	C*17	0.85	0.25	0.286	0.0332–2.453	* *
**C**	C*18	0.17	0.00	0.000	0.00	* *
**DRB1**	DRB1*3	20.86	21.23	1.024	0.8375–1.2521	* *
**DRB1**	DRB1*15	19.30	17.51	0.889	0.7188–1.0993	* *
**DRB1**	DRB1*7	12.22	13.18	1.092	0.8541–1.3966	* *
**DRB1**	DRB1*11	10.58	12.17	1.173	0.9067–1.5181	* *
**DRB1**	DRB1*13	9.99	7.55	0.737	0.5485–0.9902	**p ≤ 0.05**
**DRB1**	DRB1*4	6.86	8.05	1.191	0.8723–1.6265	* *
**DRB1**	DRB1*14	7.23	6.74	0.929	0.6729–1.2831	* *
**DRB1**	DRB1*10	5.07	6.24	1.248	0.8758–1.7792	* *
**DRB1**	DRB1*12	3.13	1.71	0.539	0.3052–0.9533	**p ≤ 0.05**
**DRB1**	DRB1*1	2.24	2.62	1.176	0.6913–2.0021	* *
**DRB1**	DRB1*8	1.12	1.21	1.083	0.5045–2.3234	* *
**DRB1**	DRB1*9	0.60	1.11	1.859	0.745–4.6389	* *
**DRB1**	DRB1*16	0.52	0.40	0.772	0.2253–2.6435	* *
**DRB1**	DRB1*5	0.22	0.00			* *
**DRB1**	DRB1*2	0.00	0.20			* *
**DRB1**	DRB1*6	0.07	0.10			* *
**DQB1**	DQB1*2	28.33	30.59	1.106	0.9237–1.3241	* *
**DQB1**	DQB1*3	26.23	26.93	1.028	0.8539–1.2386	* *
**DQB1**	DQB1*6	26.83	22.87	0.803	0.6629–0.9722	**p ≤ 0.05**
**DQB1**	DQB1*5	17.19	18.29	1.071	0.8639–1.3279	* *
**DQB1**	DQB1*4	1.12	1.22	1.083	0.5045–2.3234	* *
**DQB1**	DQB1*1	0.3	0.1	0.337	0.0376–3.0231	* *
**DQA1**	DQA1*1	44.38	41.87	0.899	0.7618–1.0615	* *
**DQA1**	DQA1*5	33.51	34.45	1.039	0.8732–1.2353	* *
**DQA1**	DQA1*2	10.79	11.89	1.112	0.858–1.4404	* *
**DQA1**	DQA1*3	7.35	9.76	1.359	1.0127–1.8242	**p ≤ 0.05**
**DQA1**	DQA1*6	2.85	1.12	0.385	0.1956–0.7562	**p ≤ 0.05**
**DQA1**	DQA1*4	1.12	0.91	0.810	0.3528–1.8575	* *

For HLA-A allele types, no significant differences were observed between the ESRD patients and the control group (**Table 1**). For HLA-B allele types, only HLA-B*40 and HLA-B*50 display significant differences in their percentage frequencies between the two groups (**Table 1**). HLA-B*40 showed a significant negative association with ESRD (OR = 0.751, P ≤ 0.05) and HLA-B*50 showed a significant positive association with ESRD (OR = 1.569, P≤ 0.05). For HLA-C allele types, again, no significant differences were observed between the ESRD patients and the control group (**Table 1**).

In the analysis of the association of HLA-DRB1, HLA-DQB1, and HLA-DQA1 with ESRD, HLA-DRB1*13, HLA-DRB1*12, HLA-DQB1*6, HLA-DQA1*3 and HLA-DQA1*6 display significant differences in their percentage frequencies between the two groups (**Table 1**). Among these significant negative association were found for HLA-DRB1*13 (OR = 0.737, P≤ 0.05), HLA-DRB1*12 (OR = 0.539, P≤ 0.05), HLA-DQB1*6 (OR = 0.803, P≤ 0.05) and HLA-DQA1*6 (OR = 0.385, P≤ 0.05). On the other hand, a significant positive association was found only for HLA-DQA1*3 (OR = 1.359, P≤ 0.05). At each locus, few alleles were only present in either the control or ESRD population. But their allele frequencies were too low to draw any conclusion.

### Effect of heterozygosity or homozygosity of the ESRD-associated HLA alleles on prevalence and age of onset of the disease

Previous works have hypothesized that HLA polymorphism at different loci could influence the susceptibility to ESRD. Here, we aimed to determine whether heterozygosity or homozygosity of the disease-associated HLA alleles at different loci could modify the prevalence of the disease. To achieve that we examined the association of different allele combinations at HLA-A, -B, -C, -DRB1, -DQB1 and DQA1 loci with ESRD. **Table 2** shows the allele combinations that displayed a significant difference in their percentage frequencies between the ESRD patients and the control group. On HLA-A locus, we identified only one allele combination, A*3_26 (a combination of HLA-A*3 and HLA-A*26), that showed significant positive association (OR = 4.488, P≤ 0.05) with ESRD. Both of these alleles had separately demonstrated a positive association with ESRD (**Table 1**); however, the differences in their percentage frequencies between control and ESRD group did not reach statistical significance. In our data, this allele combination showed the highest strength of association with ESRD.

**Table 2:** Percentage frequencies for HLA allele combinations in control and ESRD patients.

Locus	Allele Combination	Controls (% frequency)	ESRD (% frequency)	OR	CI	P value
A	A*3_26	0.30	1.37	4.4881	0.9283–21.6984	0.0488
B	B*40_51	3.55	1.56	0.4324	0.1918–0.9749	0.0434
B	B*15_40	2.93	0.98	0.3273	0.1213–0.8826	0.022
B	B*35_39	0.00	0.78	2.2787	2.1347–2.4323	0.0375
B	B*44_44	0.00	0.78	2.2787	2.1347–2.4323	0.0375
DQB1	DQB1*6_6	9.27	6.07	0.6366	0.4058–0.9985	0.0484
DRB1	DRB1*4_13	2.31	0.58	0.2489	0.0717–0.8646	0.0284

On HLA-B locus, allele combination B*40_51 showed significant negative association (OR = 0.4324, P≤ 0.05 with ESRD. In the initial analyses, B*40 showed a significant negative association with ESRD (**Table 1**). B*51 allele also showed a negative association with ESRD (**Table 1**); however, the differences in percentage frequency between control and ESRD population did not reach statistical significance. The combination B*40_15 showed a significant negative association (OR = 0.3273, P≤ 0.05) with ESRD. When analyzed separately, the B*15 allele also showed a negative association with ESRD (**Table 1**); however, its percentage frequency was not significantly different between control and ESRD population. Homozygous combination B*44_44 and heterozygous combination B*35_39 were only present in ESRD patients, but the percentage frequencies for these combinations were too low to claim their significance conclusively.

At HLA-DQB1 locus, a homozygous combination of DQB1*6_6 showed a significant negative association (OR = 0.6366, P<0.05) with ESRD. At HLA-DRB1 locus, a combination of DRB1*4_13 showed a significant negative association (OR = 0.2489, P<0.05) with ESRD.

Next, we sought to determine whether different HLA allele combinations affect the age of onset for ESRD. **S1–S6 Figs** compare the age of onset for all the identified allele combinations (percentage frequency > 0.1%) at HLA-A, -B, -C, -DRB1, -DQB1 and DQA1 loci. We observed certain differences in age of onset among individuals carrying different allele combinations. However, these differences fail to reach statistical significance.

## Discussion

Human leukocyte antigen (HLA) has been associated with a variety of renal disorders [1], and specific HLA alleles are known to confer susceptibility to multiple immune-mediated kidney diseases. Some other renal disorders–that are not conventionally classified as immune-mediated disorders–are also shown to be associated with different HLA alleles [1]. Multiple works have studied possible associations between different HLA types and ESRD [3–5]. Most of these reports have studied ethnically homogenous groups of ESRD patients that were waiting for renal transplantation. However, these works display no consistent association of different HLA alleles with ESRD. The primary aim of the presented work was to revisit the association between HLA alleles and ESRD in the Pakistani population and compare our data with previous major works. As shown above, our study also identified multiple alleles and allele combinations that showed significant associations with ESRD. Testing for significant differences within populations requires large study group sizes that withstand the necessary, multiple testing correction (MTC). HLA loci are highly polymorphic and harbor many different alleles. In the presented study, we included all the alleles that displayed the percentage frequency of >0.1%, which led to 87 allele based subgroups. The percentage frequencies of these groups were compared between ESRD patients and healthy controls. The p-values obtained from these comparisons were as low as 0.01 (DQA1*6), which was limited by the current population size and the effect strength present in this population. Using strict MTC in our approach would have rendered the moderate effects we observed insignificant. We, therefore, decided to omit MTC, which is a limitation of this study.

We also performed an extensive literature survey and collected information on significant HLA and ESRD associations from previous studies. We aimed to identify any consistent HLA associations with ESRD that can be similar across different ethnic groups. **S2 Table** shows the HLA allele types that have shown significant associations (negative or positive) with ESRD in our work or previous studies. For HLA-A locus, the HLA-A*11 allele showed a significant positive association with ESRD in three separate studies–that included study-participants from different populations [12–14]. In contrast, HLA-A*28 showed a significant negative association with ESRD in two of the previous works [15, 16]. Other HLA-A alleles also showed significant associations with ESRD. However, the association was noted in only one study, or there were contradictions in the trends of association (negative or positive) in separate works. For HLA-B locus, multiple alleles showed significant associations with ESRD. HLA-B*15 was found to be positively associated with ESRD by four different works [12, 14, 17, 18]. HLA-B*55 [14, 19] and HLA-B*53 [13, 20] were observed to be positively associated with ESRD in two studies. On the other hand, HLA-B*52 was negatively associated with ESRD in two studies [13, 21]. HLA-B*40 and HLA-B*50, which respectively showed a significant negative and positive association with ESRD in our study, showed contradictory but significant trends of associations by other works. HLA-B*18, HLA-B*39, and HLA-B*8 also showed contradictions in the trend of association (negative or positive) in separate works. Multiple alleles at HLA-C locus also showed significant associations with ESRD, but we do not find any consistent patterns among different studies.

Several HLA-DRB1 alleles also displayed consistent patterns of association with ESRD in different populations; for instance, HLA-DRB1*3, *4, and *11 all showed a significant positive association with ESRD in several separate works (**S2 Table**). HLA-DQB1*6 allele was found to be negatively associated with ESRD in our study as well as three other works. Multiple other alleles also showed significant associations with ESRD. However, these observations had certain limitations, for instance, the association was noted in only one study, or there were contradictions in the trend of association (negative or positive).

Hence, these comparisons between our data and previous works show that at least for certain alleles, the association of HLA to ESRD can be similar across different ethnic groups. It has been previously hypothesized that the association of HLA types with ESRD might be confounded by the presence of HLA associations with other diseases. For instance, a large, phenome-wide association study, showed that the HLA-DQB1*03:02 allele is associated with kidney transplantation (OR 1.4)–which was potentially related to the increased risk of and diabetic kidney disease (OR 7.1) [22]. Further studies are required to understand the association of HLA with other comorbidities.

## Supporting information

S1 Fig Effect of allele combinations at HLA-A locus on the age of onset for ESRD. The ANOVA test is used to examine significant differences among the groups, with a *P* value threshold of 0.05. *X-axis* (lower) displays different allele combinations and x-axis (upper) displays number of participants in each group.
(TIF)

S2 Fig Effect of allele combinations at HLA-B locus on the age of onset for ESRD. The ANOVA test is used to examine significant differences among the groups, with a *P* value threshold of 0.05. *X-axis* (lower) displays different allele combinations and x-axis (upper) displays number of participants in each group.
(TIF)

S3 Fig Effect of allele combinations at HLA-C locus on the age of onset for ESRD. The ANOVA test is used to examine significant differences among the groups, with a *P* value threshold of 0.05. *X-axis* (lower) displays different allele combinations and x-axis (upper) displays number of participants in each group.
(TIF)

S4 Fig Effect of allele combinations at HLA-DRB1 locus on the age of onset for ESRD. The ANOVA test is used to examine significant differences among the groups, with a *P* value threshold of 0.05. *X-axis* (lower) displays different allele combinations and x-axis (upper) displays number of participants in each group.
(TIF)

S5 Fig Effect of allele combinations at HLA-DQB1 locus on the age of onset for ESRD. The ANOVA test is used to examine significant differences among the groups, with a *P* value threshold of 0.05. *X-axis* (lower) displays different allele combinations and x-axis (upper) displays number of participants in each group.
(TIF)

S6 Fig Effect of allele combinations at HLA-DQA1 locus on the age of onset for ESRD. The ANOVA test is used to examine significant differences among the groups, with a *P* value threshold of 0.05. *X-axis* (lower) displays different allele combinations and x-axis (upper) displays number of participants in each group.
(TIF)

S1 Table Most frequent HLA alleles in Pakistani population: data from previous works. (DOCX)

S2 Table HLA alleles and ESRD. (DOCX)
