# Supplementary figures and images for: Revisiting the association between human leukocyte antigen and end-stage renal disease
Source: PLoS One. 2020 Sep 11;15(9):e0238878. doi: 10.1371/journal.pone.0238878 (PMC7485852; doi:10.1371/journal.pone.0238878)

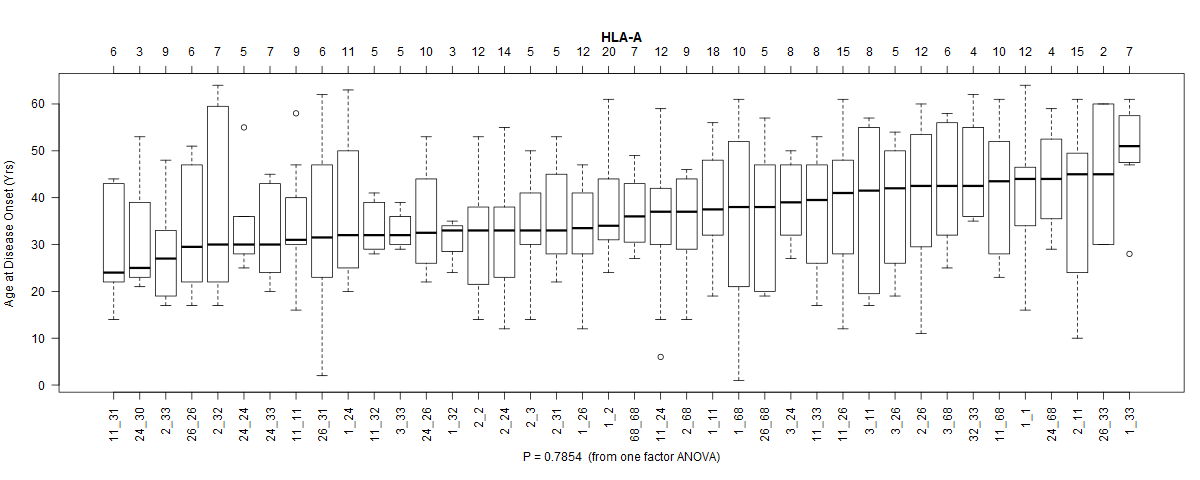

Supplement: S1 Fig — The ANOVA test is used to examine significant differences among the groups, with a P value threshold of 0.05. X-axis (lower) displays different allele combinations and x-axis (upper) displays number of participants in each group. (TIF) [file pone.0238878.s001.tif]

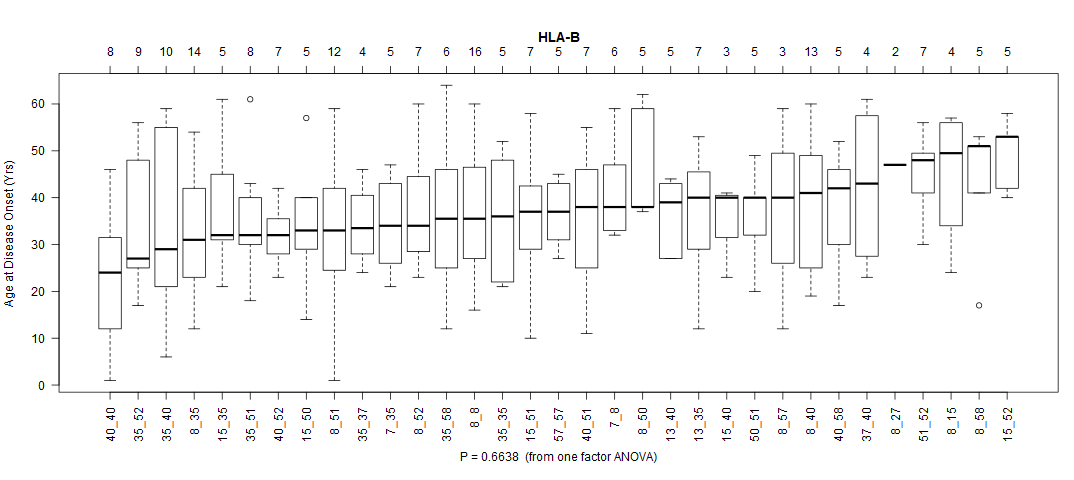

Supplement: S2 Fig — The ANOVA test is used to examine significant differences among the groups, with a P value threshold of 0.05. X-axis (lower) displays different allele combinations and x-axis (upper) displays number of participants in each group. (TIF) [file pone.0238878.s002.tif]

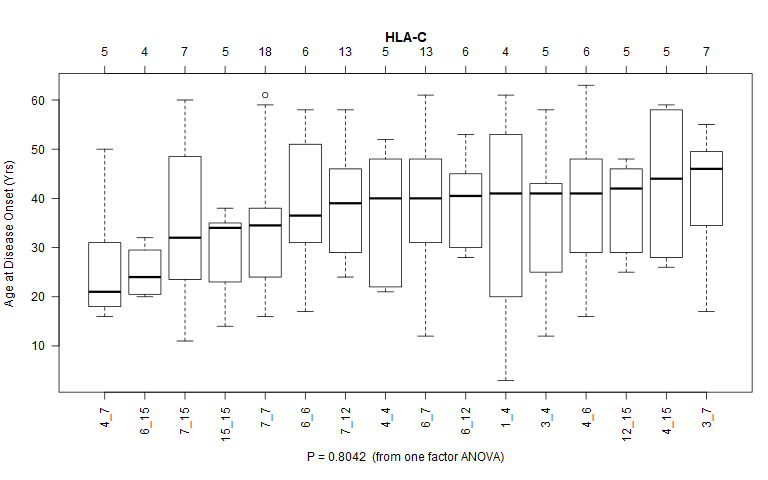

Supplement: S3 Fig — The ANOVA test is used to examine significant differences among the groups, with a P value threshold of 0.05. X-axis (lower) displays different allele combinations and x-axis (upper) displays number of participants in each group. (TIF) [file pone.0238878.s003.tif]

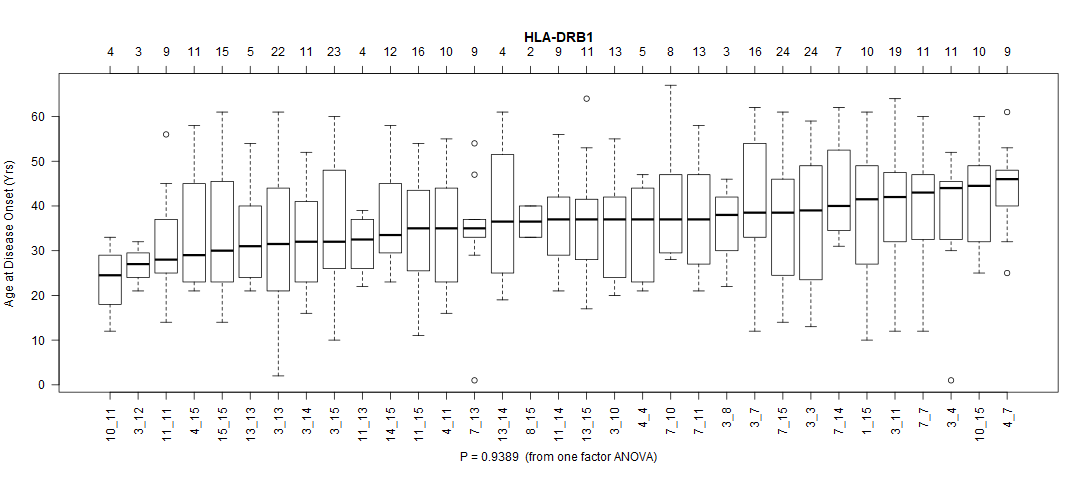

Supplement: S4 Fig — The ANOVA test is used to examine significant differences among the groups, with a P value threshold of 0.05. X-axis (lower) displays different allele combinations and x-axis (upper) displays number of participants in each group. (TIF) [file pone.0238878.s004.tif]

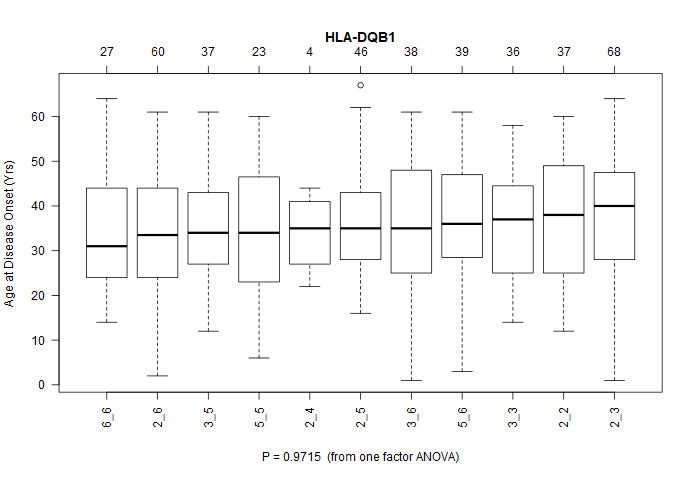

Supplement: S5 Fig — The ANOVA test is used to examine significant differences among the groups, with a P value threshold of 0.05. X-axis (lower) displays different allele combinations and x-axis (upper) displays number of participants in each group. (TIF) [file pone.0238878.s005.tif]

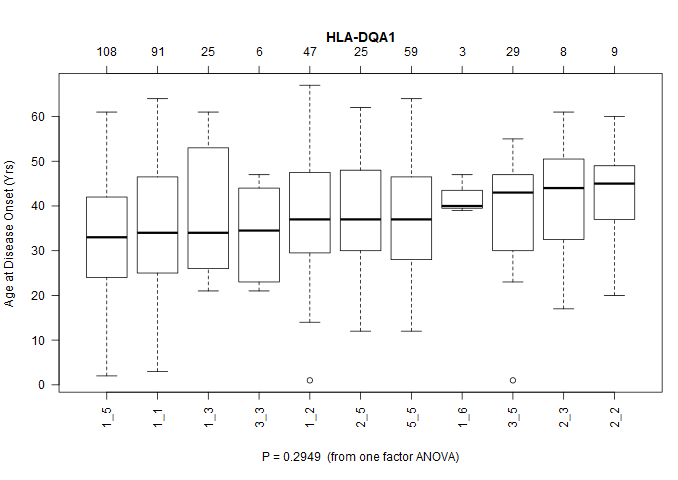

Supplement: S6 Fig — The ANOVA test is used to examine significant differences among the groups, with a P value threshold of 0.05. X-axis (lower) displays different allele combinations and x-axis (upper) displays number of participants in each group. (TIF) [file pone.0238878.s006.tif]
