# Supplementary material for: Revisiting the association between human leukocyte antigen and end-stage renal disease
Source: PLoS One. 2020 Sep 11;15(9):e0238878. doi: 10.1371/journal.pone.0238878 (PMC7485852; doi:10.1371/journal.pone.0238878)
Supplement: S1 Table — (DOCX) [file pone.0238878.s007.docx]

| **Supplementary Table S1 -** Most frequent HLA alleles in Pakistani population: data from previous works. | | | | | | | | | |
| --- | --- | --- | --- | --- | --- | --- | --- | --- | --- |
| **Reference** | | **[1]** | **[2]** | **[3]** | | | | | |
| **Ethnic Group Studied** | | Not specified | Gujjar | Baloch | Barhui | Burusho | Kalash | Pakhtoon | Sindhi |
| **n** | | 1000 | 97 | 65 | 104 | 98 | 69 | 99 | 101 |
| **HLA Loci Typed** | **A** | *02 | *02 | *11 | *11 | *33 | *02 | *02,*11 | *11 |
|  | **B** | *35 | *51 | *40 | *35 | *35 | *51 | *51 | *51 |
|  | **C** | *07 | *07 | *04 | *04 | *04 | *14 | *04 | *15 |
|  | **DRB1** | *15 | *13 | *03 | *03 | *03 | *15,*13 | *15,*13 | *03 |
|  | **DQB1** | *03 | *02 | *02 | *02 | *02,*06 | *02 | *02,*05 | *02 |

**References for Supplementary Table S1**

1. Moatter, T., et al., *Molecular analysis of human leukocyte antigen class I and class II allele frequencies and haplotype distribution in Pakistani population.* Indian journal of human genetics, 2010. **16**(3): p. 149.

2. Raza, A., et al., *HLA class I and II polymorphisms in the Gujjar population from Pakistan.* Immunological investigations, 2013. **42**(8): p. 691-700.

3. Mohyuddin, A., et al., *HLA polymorphism in six ethnic groups from Pakistan.* Tissue antigens, 2002. **59**(6): p. 492-501.
