# Supplementary material for: Revisiting the association between human leukocyte antigen and end-stage renal disease
Source: PLoS One. 2020 Sep 11;15(9):e0238878. doi: 10.1371/journal.pone.0238878 (PMC7485852; doi:10.1371/journal.pone.0238878)
Supplement: S2 Table — (DOCX) [file pone.0238878.s008.docx]

| **Supplementary Table S2: HLA alleles and ESRD -** To identify any consistent association of HLA alleles with ESRD, we performed an extensive literature survey and collected information on significant associations. The allele types that displayed significant associations in more than one study are highlighted (**Bold Letters**/Grey Cells). **Abbreviations:** P, number of ESRD patients; C, number of control subjects; N.A, Not Available. | | | | | | | | | | |
| --- | --- | --- | --- | --- | --- | --- | --- | --- | --- | --- |
| **HLA- A** | **0R** | **RR** | **CI** | **Test** | **P value** | **Association with ESRD** | **Population Type** | **Population Size** | **Study type** | **References** |
| **A*26** | N.A | 0.540 | 0.3196 - 0.9123 | Fisher’s exact | 0.0318 | Negative | Saudi Arabian | P=350, C=105 | Retrospective | [[1](#_ENREF_1)] |
|  | 7.450 | N.A | 1.29-42.92) | χ2 test | < .01 | Positive | Vietnamese | P=196, C=187 | Retrospective | [[2](#_ENREF_2)] |
| **A*11**  **A*11:01** | 1.550 |  | 1.01-2.39 | Fisher’s exact | 0.0400 | Positive | Vietnamese | P=196, C=187 | Retrospective | [[2](#_ENREF_2)] |
|  | 1.610 | 1.386 | 1.14-2.27 | χ2 test | < .015 | Positive | Mexico | P=1965,C=1361 | Retrospective | [[3](#_ENREF_3)] |
|  | *N.A* | *N.A* | 0.108–17.869 | χ2 test | <0.001 | Positive | China | P=499, C=1584 | Retrospective | [[4](#_ENREF_4)] |
| **A*30** | 0.520 | *N.A* | 0.28 -0.98 | χ2 test | 0.0450 | Negative | Yemen | P=187, C=194 | Case-control | [[5](#_ENREF_5)] |
|  | 4.820 | *N.A* | 1.13-20.47 | χ2 test | 0.0200 | Positive | Vietnamese | P=196, C=187 | Retrospective | [[2](#_ENREF_2)] |
| **A*28** | 0.120 | *N.A* | 0.01 to 1.00 | χ2 test | 0.0510 | Negative | Yemen | P=187, C=194 | Case-control | [[5](#_ENREF_5)] |
|  | 0.420 | *N.A* | 0.06–0.42 | Fisher’s exact | 0.0001 | Negative | Kuwait | P=50, C=50 | Retrospective | [[6](#_ENREF_6)] |
| A*31:01 | *N.A* | 1.480 | 0.257–8.536 | χ2 test | 0.0020 | Positive | China | P=499, C=1584 | Retrospective | [[4](#_ENREF_4)] |
| A*24 | *N.A* | *N.A* | *N.A* | Fisher’s exact | 0.0003 | Positive | China | P= 4541, C= 3744 | Retrospective | [[7](#_ENREF_7)] |
| A*9 | 0.250 | *N.A* | *N.A* | Fisher’s exact | 0.0019 | Negative | Venezuela | P=188, C=202 | Retrospective | [[8](#_ENREF_8)] |
| A*2 | 3.600 | *N.A* | *N.A* | *N.A* | 0.0050 | Positive | Iraq | P=200, C=110 | Retrospective | [[9](#_ENREF_9)] |
| A*01 | 4.720 | *N.A* | 1.32-16.87 | Fisher’s exact | 0.0200 | Positive | Vietnamese | P=196, C=187 | Retrospective | [[2](#_ENREF_2)] |
| A*25 | *N.A* | *N.A* | *N.A* | Fisher’s exact | < .01 | Positive | Vietnamese | P=196, C=187 | Retrospective | [[2](#_ENREF_2)] |
| A*78 | *N.A* | 30.310 | *N.A* | Fisher’s exact | < .01 | Positive | Brazil | P=105, C=160 | Retrospective | [[10](#_ENREF_10)] |
| **HLA-B** |  |  |  |  |  |  |  |  |  |  |
| **B*50** | 1.569 |  | 1.0328-2.383 | Fisher’s exact | 0.0405 | Positive | Pakistan | P=497, C=672 |  | **Our data** |
|  | *N.A* | 0.657 | 0.4596 -0.9386 | Fisher’s exact | 0.0260 | Negative | Saudi Arabian | P=350, C=105 | Retrospective | [[1](#_ENREF_1)] |
| **B*40**  **B*40(60)** | 0.751 |  | 0.5848-0.9649 | Fisher’s exact | 0.0250 | Negative | Pakistan | P=497, C=672 |  | **Our data** |
|  | 0.190 | *N.A* | *N.A* | Fisher’s exact | 0.0215 | Negative | Venezuela | P=188, C=202 | Retrospective | [[8](#_ENREF_8)] |
|  | *N.A* | *N.A* | *N.A* | χ2 test | -0.0130 | Positive | Turkey | P=156, C=21 | Obs. Prospective | [[11](#_ENREF_11)] |
|  | *N.A* | *N.A* | *N.A* | Fisher’s exact | 0.0008 | Positive | China | P= 4541, C= 3744 | Retrospective | [[7](#_ENREF_7)] |
| **B*15**  **B*15:01** | *N.A* | 2.588 | 1.2650 -5.2925 | Fisher’s exact | 0.0044 | Positive | Saudi Arabian | P=350, C=105 | Retrospective | [[1](#_ENREF_1)] |
|  | 1.570 | *N.A* | 1.02-2.43 | Fisher’s exact | 0.0300 | Positive | Vietnamese | P=196, C=187 | Retrospective | [[2](#_ENREF_2)] |
|  | 2.404 | *N.A* | 1.2195 - 4.7397 | Fisher’s exact | 0.0093 | Positive | Serbia | P=230, C=290 | Case-control | [[12](#_ENREF_12)] |
|  | *N.A* | 1.513 | 0.179–12.796 | χ2 test | <0.0001 | Positive | China | P=499, C=1584 | Retrospective | [[4](#_ENREF_4)] |
| **B*18** | *N.A* | 4.350 | 1.0466 -18.0795 | Fisher’s exact | 0.0277 | Positive | Saudi Arabian | P=350, C=105 | Retrospective | [[1](#_ENREF_1)] |
|  | 0.320 | *N.A* | 0.17–0.59 | Fisher’s exact | 0.0001 | Negative | Kuwait | P=50, C=50 |  | [[6](#_ENREF_6)] |
|  | 1.630 | *N.A* | 1.17-2.27 | χ2 test | <0.05 | Positive | Mexico | P=1965,C=1361 | Retrospective | [[3](#_ENREF_3)] |
| **B*39**  **B*39:05** | *N.A* | 0.300 | 0.1319 - 0.6821 | Fisher’s exact | 0.0076 | Negative | Saudi Arabian | P=350, C=105 | Retrospective | [[1](#_ENREF_1)] |
|  | *N.A* | 2.625 | 2.144–3.215 | χ2 test | 0.0320 | Positive | China | P=499, C=1584 | Retrospective | [[4](#_ENREF_4)] |
| B*49 | *N.A* | 4.500 | 1.0844 -18.6739 | Fisher’s exact | 0.0181 | Positive | Saudi Arabian | P=350, C=105 | Retrospective | [[1](#_ENREF_1)] |
| B*16 | 0.110 |  | 0.00 -2.11 | χ2 test | 0.0480 | Negative | Yemen | P=187, C=194 | Case-control | [[5](#_ENREF_5)] |
| **B*55**  **B*55:05** | *N.A* | *N.A* | *N.A* | Fisher’s exact | 0.0002 | Positive | China | P= 4541, C= 3744 | Retrospective | [[7](#_ENREF_7)] |
|  | *N.A* | 1.500 | 0.276–8.147 | χ2 test | 0.0100 | Positive | China | P=499, C=1584 | Retrospective | [[4](#_ENREF_4)] |
| B*54 | *N.A* | *N.A* | *N.A* | Fisher’s exact | 0.0002 | Positive | China | P= 4541, C= 3744 | Retrospective | [[7](#_ENREF_7)] |
| **B*8** | 1.420 | *N.A* | 1.02-2.01 | χ2 test | <0.05 | Positive | Mexico | P=1965,C=1361 | Retrospective | [[3](#_ENREF_3)] |
|  | 0.730 | *N.A* | 0.29–1.84 | Fisher’s exact | 0.0010 | Negative | Kuwait | P=50, C=50 | Retrospective | [[6](#_ENREF_6)] |
| B*12 | 0.160 | *N.A* | *N.A* | Fisher’s exact | 0.0001 | Negative | Venezuela | P=188, C=202 | Retrospective | [[8](#_ENREF_8)] |
| B*17 | 0.150 | *N.A* | *N.A* | Fisher’s exact | 0.0039 | Negative | Venezuela | P=188, C=202 | Retrospective | [[8](#_ENREF_8)] |
| B*38 | 2.420 | *N.A* | *N.A* | Fisher’s exact | 0.0322 | Positive | Venezuela | P=188, C=202 | Retrospective | [[8](#_ENREF_8)] |
| B*48 | 0.080 | *N.A* | *N.A* | Fisher’s exact | 0.0307 | Negative | Venezuela | P=188, C=202 | Retrospective | [[8](#_ENREF_8)] |
| B*53 | 2.570 | *N.A* | *N.A* | Fisher’s exact | 0.0211 | Positive | Venezuela | P=188, C=202 | Retrospective | [[8](#_ENREF_8)] |
|  | 1.850 | *N.A* | 1.07-3.20 | χ2 test | <0.05 | Positive | Mexico | P=1965,C=1361 | Retrospective | [[3](#_ENREF_3)] |
| B*62 | 3.750 | *N.A* | *N.A* | Fisher’s exact | 0.0465 | Positive | Venezuela | P=188, C=202 | Retrospective | [[8](#_ENREF_8)] |
| **B*52** | *N.A* | *N.A* | *N.A* | χ2 test | 0.0130 | Negative | Turkey | P=156, C=21 | Obs. Prospective | [[11](#_ENREF_11)] |
|  | 0.730 | *N.A* | 0.55-0.97 | χ2 test | <0.05 | Negative | Mexico | P=1965,C=1361 | Retrospective | [[3](#_ENREF_3)] |
| B*58 | *N.A* | *N.A* | *N.A* | χ2 test | 0.0160 | Negative | Turkey | P=156, C=21 | Obs. Prospective | [[11](#_ENREF_11)] |
| B*35 | 3.900 | *N.A* | *N.A* | *N.A* | 0.0330 | Positive | Iraq | P=200, C=110 | Retrospective | [[9](#_ENREF_9)] |
| B*07 | 0.475 | *N.A* | 0.277-0.814 | χ2 test | 0.0100 | Negative | Vietnamese | P=196, C=187 | Retrospective | [[2](#_ENREF_2)] |
| B*13 | 0.420 | *N.A* | 0.19-0.91 | Fisher’s exact | 0.0200 | Negative | Vietnamese | P=196, C=187 | Retrospective | [[2](#_ENREF_2)] |
| B*57 | 13.000 | *N.A* | 3.35-50.32 | χ2 test | < .001 | Positive | Vietnamese | P=196, C=187 | Retrospective | [[2](#_ENREF_2)] |
| B*56 | *N.A* | *N.A* | *N.A* | Fisher’s exact | 0.0400 | Positive | Vietnamese | P=196, C=187 | Retrospective | [[2](#_ENREF_2)] |
| B*14 | *N.A* | 29.900 | *N.A* | Fisher’s exact | <0.01 | Positive | Brazil | P=105, C=160 | Retrospective | [[10](#_ENREF_10)] |
| **HLA-C** |  |  |  |  |  |  |  |  |  |  |
| C*06:02 | *N.A* | 0.826 | 0.056–12.299 | χ2 test | 0.0290 | Negative | China | P=499, C=1584 | Retrospective | [[4](#_ENREF_4)] |
| C*07:01 | *N.A* | 0.520 | 0.098–2.752 | χ2 test | 0.0320 | Negative | China | P=499, C=1584 | Retrospective | [[4](#_ENREF_4)] |
| C*03 | *N.A* | 0.000 | 0.0–0.72 | χ2 test | 0.0060 | Negative | Yemen | *N.A* | Retrospective | [[13](#_ENREF_13)] |
| C*01 | *N.A* | *N.A* | *N.A* | Fisher accuracy | 0.0200 | Positive | Bosnia | P=186, C=50 | Retrospective | [[14](#_ENREF_14)] |
| C*12 | *N.A* | *N.A* | *N.A* | Fisher accuracy | 0.0010 | Negative | Bosnia | P=186, C=50 | Retrospective | [[14](#_ENREF_14)] |
| C*04 | *N.A* | *N.A* | *N.A* | χ2 test | 0.0330 | Positive | Turkey | P=156, C=21 | Obs. Prospective | [[11](#_ENREF_11)] |
| C*05 | *N.A* | *N.A* | *N.A* | χ2 test | 0.0410 | Positive | Turkey | P=156, C=21 | Obs. Prospective | [[11](#_ENREF_11)] |
| **HLA-DRB1** |  |  |  |  |  |  |  |  |  |  |
| **DRB1*12**  **DRB1*12:02** | 0.539 | *N.A* | 0.3052-0.9533 | Fisher’s exact | 0.0329 | Negative | Pakistan | P=497, C=672 |  | **Our Data** |
|  | *N.A* | *N.A* | *N.A* | Fisher accuracy | 0.0280 | Positive | Turkey | P=156, C=21 | Obs. Prospective | [[11](#_ENREF_11)] |
|  | *N.A* | 1.384 | 0.130–14.680 | χ2 test | <0.004 | Positive | China | P=499, C=1584 | Retrospective | [[4](#_ENREF_4)] |
| **DRB1*13** | 0.737 | *N.A* | 0.5485-0.9902 | Fisher’s exact | 0.0476 | Negative | Pakistan | P=497, C=672 |  | **Our Data** |
|  | *N.A* | *N.A* | *N.A* | Fisher accuracy | 0.0300 | Negative | Bosnia | P=186, C=50 | Retrospective | [[14](#_ENREF_14)] |
| **DRB1*3**  **DR*3**  **DRB1*03:01** | *N.A* | 1.650 | 1.0762 -2.5296 | Fisher’s exact | 0.0173 | Positive | Saudi Arabian | P=350, C=105 | Retrospective | [[1](#_ENREF_1)] |
|  | 1.950 | *N.A* | 1.085–3.510 | Fisher’s exact | 0.0310 | Positive | Taiwan | P= 141, C= 190 | Retrospective | [[15](#_ENREF_15)] |
|  | *N.A* | 1.358 | 0.193–9.555 | χ2 test | 0.0180 | Positive | China | P=499, C=1584 | Retrospective | [[4](#_ENREF_4)] |
| **DRB1*4**  **DRB1*04:03**  **DRB1*04:04**  **DRB1*04:05**  **DRB1*0407** | *N.A* | *N.A* | *N.A* | χ2 test | 0.0001 | Positive | China | P= 4541, C= 3744 | Retrospective | [[7](#_ENREF_7)] |
|  | *N.A* | 1.648 | 1.0395 - 2.6138 | Fisher’s exact | 0.0325 | Positive | Serbia | P=230, C=290 | Case-control | [[12](#_ENREF_12)] |
|  | 1.110 | *N.A* | 1.002-1.23 | χ2 test | <0.05 | Positive | Mexico | P=1965,C=1361 | Retrospective | [[3](#_ENREF_3)] |
|  | *N.A* | 1.683 | 0.397–7.141 | χ2 test | 0.0030 | Positive | China | P=499, C=1584 | Retrospective | [[4](#_ENREF_4)] |
|  | *N.A* | 1.509 | 0.377-6.037 | χ2 test | 0.0460 | Positive | China | P=499, C=1584 | Retrospective | [[4](#_ENREF_4)] |
|  | *N.A* | 1.284 | 0.135–12.245 | χ2 test | 0.0140 | Positive | China | P=499, C=1584 | Retrospective | [[4](#_ENREF_4)] |
|  | *N.A* | 0.100 | 0.02– 0.41 | χ2 test | 0.0020 | Negative | Mexico | P= 42, C= 101 | Retrospective | [[16](#_ENREF_16)] |
| **DRB1*11**  **DR*11**  **DR*11 (5)**  **DRB1*11:01** | 1.370 |  | 1.06-1.78 | χ2 test | <0.06 | Positive | Mexico | P=1965,C=1361 | Retrospective | [[3](#_ENREF_3)] |
|  | 2.110 |  | 1.118–3.970 | Fisher’s exact | 0.0300 | Positive | Taiwan | P= 141, C= 190 | Retrospective | [[15](#_ENREF_15)] |
|  | *N.A* | 18.870 | *N.A* | Fisher’s exact | <0.01 | Positive | Brazil | P=105, C=160 | Retrospective | [[10](#_ENREF_10)] |
|  | 0.440 | *N.A* | 0.27–0.69 | Fisher’s exact | 0.0007 | Negative | Kuwait | P=50, C=50 | Retrospective | [[6](#_ENREF_6)] |
|  | *N.A* | 1.478 | 0.183–11.914 | χ2 test | <0.003 | Positive | China | P=499, C=1584 | Retrospective | [[4](#_ENREF_4)] |
| **DRB1*15:01**  **DRB1*1502** | *N.A* | 0.714 | 0.054–9.442 | χ2 test | 0.0010 | Negative | China | P=499, C=1584 | Retrospective | [[4](#_ENREF_4)] |
|  | *N.A* | 6.100 | 1.4 –51 | χ2 test | 0.0200 | Positive | Mexico | P= 42, C= 101 | Retrospective | [[16](#_ENREF_16)] |
| DR*7 | 3.610 | *N.A* | 1.062–12.284 | Fisher’s exact | 0.0360 | Positive | Taiwan | P= 141, C= 190 | Retrospective | [[15](#_ENREF_15)] |
| **DR*8**  **DRB1*8** | 0.400 | *N.A* | 0.186–0.880 | Fisher’s exact | 0.0260 | Negative | Taiwan | P= 141, C= 190 | Retrospective | [[15](#_ENREF_15)] |
|  | 0.820 | *N.A* | 0.75-0.92 | χ2 test | <0.08 | Negative | Mexico | P=1965,C=1361 | Retrospective | [[3](#_ENREF_3)] |
| DRB1*10 | 0.547 | *N.A* | 0.314-0.954 | χ2 test | 0.0300 | Negative | Vietnamese | P=196, C=187 | Retrospective | [[2](#_ENREF_2)] |
| DRB1*14 | 2.250 | *N.A* | 1.11-4.55 | χ2 test | 0.0200 | Positive | Vietnamese | P=196, C=187 | Retrospective | [[2](#_ENREF_2)] |
| DRB1*17 | 3.910 | *N.A* | 2.96-5.17 | χ2 test | <0.07 | Positive | Mexico | P=1965,C=1361 | Retrospective | [[3](#_ENREF_3)] |
| DRB1*9 | 0.043 | *N.A* | 0.005-0.3224 | χ2 test | <0.09 | Negative | Mexico | P=1965,C=1361 | Retrospective | [[3](#_ENREF_3)] |
| DRB1*16 | 0.090 | *N.A* | 0.06-0.15 | χ2 test | <0.10 | Negative | Mexico | P=1965,C=1361 | Retrospective | [[3](#_ENREF_3)] |
| **HLA-DQB1** |  |  |  |  |  |  |  |  |  |  |
| **DQB1*6**  **DQB1*6**  **DQB1*06:02**  **DQB1*06:09** | 0.803 | *N.A* | 0.6629-0.9722 | Fisher’s exact | 0.0262 | Negative | Pakistan | P=497, C=672 |  | **Our Data** |
|  | *N.A* | *N.A* | *N.A* | χ2 test | 0.0280 | Negative | Bosnia | P=186, C=50 | Retrospective | [[14](#_ENREF_14)] |
|  | *N.A* | 0.554 | 0.046–6.713 | χ2 test | <0.004 | Negative | China | P=499, C=1584 | Retrospective | [[4](#_ENREF_4)] |
|  | *N.A* | 0.632 | 0.087–4.578 | χ2 test | 0.0220 | Negative | China | P=499, C=1584 | Retrospective | [[4](#_ENREF_4)] |
| DQB1*02:01 | *N.A* | 1.399 | 0.201–9.746 | χ2 test | 0.0090 | Positive | China | P=499, C=1584 | Retrospective | [[4](#_ENREF_4)] |
| **DQB1*03**  **DQB1*03:02** | *N.A* | *N.A* | *N.A* | χ2 test | 0.0320 | Positive | Turkey | P=156, C=21 | Obs. Prospective | [[11](#_ENREF_11)] |
|  | *N.A* | 1.248 | 0.133–11.737 | χ2 test | 0.0350 | Positive | China | P=499, C=1584 | Retrospective | [[4](#_ENREF_4)] |
| DQB1*04:01 | *N.A* | 1.282 | 0.138–11.925 | χ2 test | 0.0170 | Positive | China | P=499, C=1584 | Retrospective | [[4](#_ENREF_4)] |
| DQB1*0501 | *N.A* | 2.900 | 1.5–37 | χ2 test | 0.0070 | Positive | Mexico | P= 42, C= 101 | Retrospective | [[16](#_ENREF_16)] |
| **HLA-DQA1** |  |  |  |  |  |  |  |  |  |  |
| **DQA1*3**  **DQA*3** | 1.359 | *N.A* | 1.0127-1.8242 | Fisher’s exact | 0.0484 | Positive | Pakistan | P=497, C=672 |  | **Our Data** |
|  | 0.860 | *N.A* | 0.74-0.99 | χ2 test | 0.0500 | Negative | Mexico | P=1965,C=1361 | Retrospective | [[3](#_ENREF_3)] |
| DQA1*6 | 0.385 | *N.A* | 0.1956-0.7562 | Fisher’s exact | 0.0116 | Negative | Pakistan | P=497, C=672 |  | **Our Data** |
| DQA*4 | 0.790 | *N.A* | 0.67-0.93 | χ2 test | 0.0500 | Negative | Mexico | P=1965,C=1361 | Retrospective | [[3](#_ENREF_3)] |

**References for Supplementary Table S2**

1. Hamdi, N.M., F.H. Al-Hababi, and A.E. Eid, *HLA class I and class II s with ESRD in Saudi Arabian population.* PloS one, 2014. **9**(11).

2. Hieu, H.T., N.T. Ha, and T.H. Nghi.  *of Human Leukocyte Antigen Haplotypes With End-Stage Renal Disease in Vietnamese Patients Prior to First Transplantation*. in *Transplantation proceedings*. 2019. Elsevier.

3. Hernández-Rivera, J.C.H., et al., *Most common HLA alleles associated with risk and/or protection in chronic kidney disease of undetermined etiology.* Gac Med Mex, 2019. **155**: p. 223-227.

4. Pan, Q., et al., *A single center study of protective and susceptible HLA alleles and haplotypes with end-stage renal disease in China.* Human Immunology, 2019. **80**(11): p. 943-947.

5. Nassar, M.Y., et al., *Human Leukocyte Antigen Class I and II Variants in Yemeni Patients with Chronic Renal Failure.* Iranian Journal of Immunology, 2017. **14**(3): p. 240-249.

6. Mosaad, Y.M., et al.,  *between Human Leukocyte Antigens (HLA-A, -B, and -DR) and end-stage renal disease in Kuwaiti patients awaiting transplantation.* Renal failure, 2014. **36**(8): p. 1317-1321.

7. Cao, Q., et al., *HLA polymorphism and susceptibility to end-stage renal disease in Cantonese patients awaiting kidney transplantation.* PloS one, 2014. **9**(3).

8. Rivera, S., et al., *HLA class I with progression to end-stage renal disease in patients from Zulia, Venezuela.* Inmunologia, 2012. **31**(2): p. 37-42.

9. Al-Taie, L.H., et al., *Frequency of HLA-A and B antigens in Iraqi patients with end-stage renal disease preparing for transplantation.* Iraqi Academic Scientific Journal, 2012. **11**(supplement): p. 642-648.

10. Crispim, J., et al. *HLA polymorphisms as incidence factor in the progression to end-stage renal disease in Brazilian patients awaiting kidney transplant*. in *Transplantation proceedings*. 2008. Elsevier.

11. Kodaz, H., D. Akdeniz, and K. Cengiz,  *Between Human Leukocyte Antigens and Chronic Renal Disease.* EURASIAN JOURNAL OF MEDICAL INVESTIGATION, 2017. **1**(1): p. 1-5.

12. Ademović-Sazdanić, D. and S. Vojvodić, *Human leukocyte antigen polymorphisms as susceptibility risk factors for end stage renal disease.* Genetika, 2019. **51**(2): p. 607-617.

13. Nassar, M.Y., H.A. Al-Shamahy, and H.A. Masood, *The between Human Leukocyte Antigens and Hypertensive End-Stage Renal Failure among Yemeni Patients= العلاقة بين أنواع مستضدات الكريات البيض البشرية في مرض المرحلة الأخيرة من الفشل الكلوي المصاحب لارتفاع ضغط الدم بين المرضى اليمنيين.* Sultan Qaboos University Medical Journal, 2015. **22**(2617): p. 1-9.

14. Fejzić, E., et al., *HLA Genotyping in Patients with End-Stage Renal Disease Waiting For Cadaveric Renal Transplantation in Federation of Bosnia and Herzegovina.* Open access Macedonian journal of medical sciences, 2017. **5**(1): p. 1.

15. Dai, C.-S., et al.,  *between human leucocyte antigen subtypes and risk of end stage renal disease in Taiwanese: a retrospective study.* BMC nephrology, 2015. **16**(1): p. 177.

16. Pérez-Luque, E., et al., *Contribution of HLA class II genes to end stage renal disease in mexican patients with type 2 diabetes mellitus.* Human Immunology, 2000. **61**(10): p. 1031-1038.
